# Mercury exposure and Minamata disease in Brazil: evidence or alarm?

**DOI:** 10.1055/s-0046-1817051

**Published:** 2026-04-28

**Authors:** Marcos Vinicius Sousa Varão, Guilherme Nobre Nogueira, Gunter Gerson

**Affiliations:** 1 Universidade Federal do Ceará, Fortaleza CE, Brazil.; 2 Universidade Federal do Ceará, Faculdade de Medicina, Departamento de Patologia e Medicina Legal, Fortaleza CE, Brazil.

Dear editor,

I have read with great interest the article “A Brazilian Minamata Disease? Neurologists must be aware of mercury exposure and intoxication,” by Maximiano-Alves et al., 1 recently published in *Arquivos de Neuro-Psiquiatria* . The topic is of undeniable relevance, especially considering the alarming expansion of mercury exposure in the Amazon region and the need for neurologists to recognize its clinical implications. Nevertheless, there are some topics that need further discussion.

The study is particularly valuable for its focus on indigenous and riverside populations, which are among the most vulnerable and have a high prevalence of neurological and motor deficits associated with mercury. The detailed list of more than 250 neurological symptoms related to mercury exposure serves as a practical and essential guide for neurologists, covering everything from cognitive impairment and cerebellar ataxia to psychiatric disorder and peripheral neuropathy. 1

These findings reinforce the need for greater clinical and diagnostic awareness, as mercury's toxicity to neurological development is well-documented. 2 Research on the effects of methylmercury exposure in children, 2 3 for example, has shown a significant impact on neurocognitive function and toxicity to the developing brain. Case studies from Minamata, Japan, illustrate the devastating consequences of chronic methylmercury exposure, 3 with research showing that the poisoning can lead to structural changes in the brain and neurocognitive impairment. 4

The article raises a provocative question in its title—“A Brazilian Minamata disease?”—implying a potential public health crisis of similar magnitude to the original Minamata episode in Japan. However, the paper does not provide concrete epidemiological evidence to substantiate this comparison. There is no documentation of a widespread outbreak with a consistent pattern of neurological or systemic symptoms that would support the existence of a parallel epidemic; instead, most available data point toward underreporting and diagnostic gaps, rather than a confirmed large-scale event.

Moreover, the article intertwines epidemiological, environmental, and neurological aspects without a clearly-delineated conceptual framework. This multidisciplinary approach is commendable; yet. it results in conceptual dispersion and limited analytical coherence. The narrative transitions abruptly from macroenvironmental phenomena, such as illegal mining activities and mercury trafficking, to highly-specific neurotoxicological and clinical descriptions. Structuring the discussion into distinct sections, one dedicated to pathophysiological and clinical mechanisms, and another addressing public health implications and policy responses, would enhance clarity, logical flow, and the overall scientific impact of the work.

The research does not limit its scope to mercury exposure resulting from illegal gold mining; it also highlights other relevant sources of contamination, such as the use of dental amalgams, the presence of mercury in imported cosmetics, and the consumption of fish and seafood. This broader perspective demonstrates a holistic and comprehensive understanding of the issue, acknowledging the multifactorial nature of mercury exposure in Brazil.

In conclusion, “A Brazilian Minamata Disease?” is a relevant and timely contribution that successfully calls attention to mercury-related neurotoxicity in Brazil. Nonetheless, the article would benefit from a structured synthesis of evidence, and a more critical interpretation of available data. Refining these aspects would elevate its value as a scientific reference and strengthen its role in guiding future studies.

## References

[1] Maximiano-Alves G Dantas ELdS Crespo-Lopez M E Nascimento JLMd A Brazilian Minamata disease? Neurologists must be aware of mercury exposure and intoxication Arq Neuropsiquiatr 2025 83 09 1 10 10.1055/s-0045-1811622 PMC12417040 40921410

[2] Grandjean P Landrigan P J Neurobehavioural effects of developmental toxicity Lancet Neurol 2014 13 03 330 338 10.1016/S1474-4422(13)70278-3 24556010 PMC4418502

[3] Yorifuji T Yamamura Y Nagano I Kado Y Shigeoka S Fujino T Prenatal methylmercury poisoning and neurocognitive impairment in Minamata Sci Total Environ 2025 985 179743 10.1016/j.scitotenv.2024.179743 40440848

[4] Hirai T Abe O Nakamura M Brain structural changes in patients with chronic methylmercury poisoning in Minamata Brain Res 2023 1805 148278 10.1016/j.brainres.2023.148278 36775085

